# Effect of the Interface on the Compressibility of Substances with Spherical Nano-Inhomogeneities on the Example of Al/C_60_

**DOI:** 10.3390/nano12122045

**Published:** 2022-06-14

**Authors:** Viktor Reshetniak, Olga Reshetniak, Artemiy Aborkin, Vladimir Nederkin, Anatoliy Filippov

**Affiliations:** 1 State Research Center of the Russian Federation, Troitsk Institute for Innovation and Fusion Research, Troitsk 108840, Russia; obr@triniti.ru (O.R.); fav@triniti.ru (A.F.); 2 Vladimir State University named after Alexander and Nikolay Stoletov, Vladimir 600000, Russia; aborkin@vlsu.ru; 3 Joint Institute for High Temperatures, Russian Academy of Sciences, Moscow 125412, Russia; 4 Moscow Institute of Physics and Technology, Dolgoprudny 141701, Russia; 5 Faculty of Physics and Technology, Donetsk National University, Donetsk 83001, Donetsk People’s Republic, Ukraine; vova.nederkin@gmail.com

**Keywords:** compressibility, nano-inhomogeneity, interface, molecular dynamics

## Abstract

The paper examines the compressibility of media with nano-inhomogeneities using the example of an aluminum melt and C_60_ fullerenes immersed in it. The results of molecular dynamics simulations indicate a significant effect of the interface on the effective compressibility of a heterogeneous medium. It is found that the application of the rule of mixture for the Al/C_60_ system results in an incorrect qualitative picture of the dependence of compressibility on the concentration of fullerenes. To explain this effect, an analytical model is proposed that takes into account the reduction in distances between atoms of different components during compression. The model makes it possible to estimate the effective mechanical characteristics of a liquid with nano-inhomogeneities within the framework of the mechanical approach, and correctly predicts the nature of the change in the dependence of compressibility on concentration.

## 1. Introduction

The problem of creating high-modulus nanoparticle-reinforced aluminum-based composites with enhanced mechanical properties has attracted considerable attention of researchers in the past decades [1,2,3]. Due to high mechanical stiffness, carbon nanostructures and, in particular, C_60_ fullerenes seem to be especially promising [4].

Al/C_60_ composites have been developed and examined in [5,6,7,8,9,10,11,12,13,14]. Theoretical studies of the dependence of energy on strain have made it possible to assess the values of the “bulk modulus” and “Young’s modulus” of fullerenes, which are about 800 GPa and 2000 GPa, respectively [15,16,17,18]. Estimates of the average elastic moduli of Al/C_60_ composites by the rule of mixture (ROM) predict an increase in the stiffness with increasing fullerene concentration. Recall that, according to ROM, average elastic moduli and elastic compliance of the composite are calculated by averaging over the components, whose concentrations are used as weight coefficients [19].

Experimental studies show that the mechanical properties of Al/C_60_ composites strongly depend on the manufacturing technology. Among other reasons for this dependence, one should single out the porosity of composite samples [6], phase transformations during high-temperature annealing, and inhomogeneous distribution of the filler in the matrix [5,8,9]. Material synthesis technologies that allow one to exclude these effects are described in detail in [7,9,12,13,14], where, after composites were synthesized, their mechanical properties, including the values of elastic moduli, were investigated. A significant increase in Vickers hardness and tensile strength of composites is most often observed by researchers. The use of the empirical dependence of the hardness of materials on the shear modulus *G* and compression modulus *B* (see paper [20]) suggests an increase in the *G*/*B* ratio. An analysis of the tension diagrams presented in [7,11] demonstrates an increase in Young’s modulus in the composite material compared to that in the original one. At the same time, the ultrasonic study of the elastic characteristics of composites [7] indicates a decrease in the bulk modulus of the composite at virtually the same (within the measurement error) value of the shear modulus.

The effect of reducing the overall compression modulus of the composite when high-modulus nanoparticles are incorporated into the matrix seems to be somewhat unexpected and deserves a detailed theoretical study. Note that the ROM obtained by neglecting the elastic interactions between inhomogeneities in a solid, as well as more general models that allow this effect to be taken into account, are usually based on the assumption that the interface is ideal [21]. When use is made of inhomogeneities up to 10 nm in size, the number of atoms at the interfaces is comparable to the number of atoms in the bulk of the substance, and the contribution of the energy of these atoms to the free energy of the system turns out to be significant [22,23]. Note also that, for nanoparticles whose radius is comparable with interatomic distances (in particular, fullerenes), such concepts as volume, density, interfacial surface, and elastic modulus are poorly defined, and therefore the applicability of continuum models for nanocomposites reinforced with such particles raises questions.

In this work, we study of the effect of the interface on the compressibility of the Al/C_60_ system by using the molecular dynamics (MD) simulation. To simplify the formulation of the problem, we consider an aluminum melt, which makes it possible to remove questions about the initial location of aluminum atoms near the conditional interfacial surface. This simplification allows us to study the effect of the interface on the compressibility of systems with a low concentration of inhomogeneities, in which the interaction of particles through elastic fields is negligibly small. The model assumes the absence of shear deformations in the system and does not answer questions about the distribution of elastic stresses in a solid. The study of the influence of nano-inhomogeneities on the distribution of stresses and deformation of solids will be presented elsewhere. Thus, the present work is aimed at studying the applicability of continuum models of mechanics to such systems. Note that the examination of the compressibility of the Al/C_60_ system with the aluminum melt can also be useful in view of the widespread use of methods for consolidating powder mixtures, associated with pulsed action on a substance with concomitant melting and crystallization [24,25,26].

## 2. Molecular Dynamics Simulation

To describe interatomic interactions, we relied on a hybrid potential with different interaction models for various pairs of atoms. For a pair of Al–Al atoms, use was made of the embedded atom model with the parametrization from Ref. [27], which provides a high accuracy in calculating the compressibility and surface tension coefficient of the aluminum melt [28]. Carbon atoms interact with each other via the Tersoff potential [29], in which the parametrization proposed in [30] was used to improve the accuracy of graphene modeling. To take into account the Al–C interaction, the Lennard–Jones potential was employed:

(1) $$E=4\epsilon \left[{\left(\frac{\sigma }{r}\right)}^{12}-{\left(\frac{\sigma }{r}\right)}^{6}\right].$$

The values of potential parameters (1), $\epsilon =5.2\times 10^{-2}$ eV, and σ=2.7 Å were borrowed from [31], where the potential (1) was parameterized according to the results of ab initio investigation of the interaction of carbon and aluminum atoms at the Al/C_60_ interface. Despite the existing drawbacks, a detailed discussion of which can be found in [31], the potential makes it possible to reproduce with high accuracy the experimental temperature dependence of the Al(111) surface coating density with C_60_ molecules, obtained in [32] by temperature-programmed desorption.

**Figure 1:**
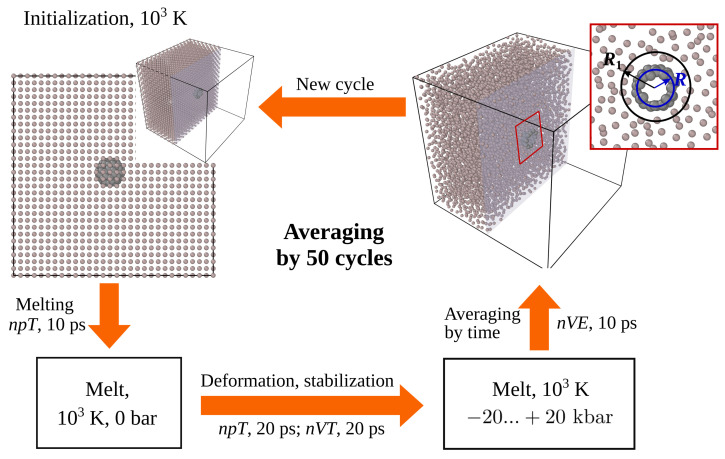
Schematic of the MD simulation procedure.

The MD calculations were performed using the LAMMPS software package [33] at a temperature of 1000 K, according to the scheme in Figure 1.

A spherical cavity was cut out in the center of the cubic computational domain containing aluminum atoms, in which the fullerene C_60_ was located. The initial parameters of the crystal cell and the arrangement of atoms were set in accordance with the structure of the aluminum crystal [34]. Systems with different mass fractions of fullerenes were studied by varying the size of the computational domain and the number of aluminum atoms. To create the initial atomic configuration, we used in this work cubic aluminum supercells N×N×N,(N=7,8,9,11,15) with one C_60_ molecule, while the mass fraction of carbon, *μ*, varied in the range from 0.2 mass% to 2.0 mass%. The cavity radius was calculated by the formula $R_{1}=R_{0}+\sigma$, where $R_{0}=3.6612$ Å is the fullerene radius in the absence of interaction with aluminum; it is defined as the average distance between the center of mass and carbon atoms at a given temperature. The integration of the equations of motion was performed with a step of 0.2 fs, the thermostat relaxation time was set equal to 100 fs, the barostat relaxation time was 1 ps, and the center of mass of the nanoparticle was fixed.

Relaxation involved two stages. First, the system was stabilized at 1000 K and zero pressure for 10 ps. In this case, aluminum was melted and an equilibrium state of the melt was established. Then, the computational cell was compressed or stretched, and the system was again stabilized at a constant volume for 20 ps. After relaxation, the thermostat was turned off and the averaged values of the nanoparticle radius *R*, the melt boundary *R*_1_ (see Figure 1), the pressure on the melt from the boundaries of the computational domain, and the radial distribution of the aluminum density relative to the center of mass of the nanoparticle, *n*(*r*), were calculated. Averaging was performed using data from 50 statistically independent calculations, in each of which the parameter values were averaged over a time interval of 10 ps.

In studying the radial distribution of the melt density, the computational domain was divided into concentric spherical surfaces, the center of which coincided with the center of mass of the fullerene. The division step was approximately 0.1 Å. An analysis of the temperature and local order parameters *q*_4_ and *q*_6_ [35,36], as well as the aluminum density radial distribution function, suggests the absence of processes associated with the crystallization or vitrification of aluminum during calculations.

It is known from the theory of capillarity that, in a liquid with a spherical inhomogeneity of radius *R*, the pressure on different sides of the interface is different. The inhomogeneity limited by the interface is subject to the action of the Laplace pressure, while, in the liquid volume, the pressure is uniformly distributed everywhere, except for the region of a small volume near the interface [37]. The thickness of this region, *d*, is comparable in magnitude with the interatomic distances, and at R≫d it can be assumed that the function of the spatial distribution of pressure changes abruptly on the interfacial surface. In the model in question, an approximation like this is inapplicable, and the fraction of aluminum deformed by surface tension forces is significant. To calculate the effective compressibility of an inhomogeneous medium, it is necessary to determine the dependence of the volume on the pressure acting on the substance from the boundaries of the computational domain, and the contribution of the Laplace pressure should be excluded. Therefore, in the MD simulation, the pressure was calculated with allowance for only the atoms located near the boundaries of the computational domain. The distance to the boundaries was 0.05 of the size of the computational domain; in this case, at least 350 atoms participated in the averaging. The components of the local stress tensor were calculated according to [38].

**Figure 2:**
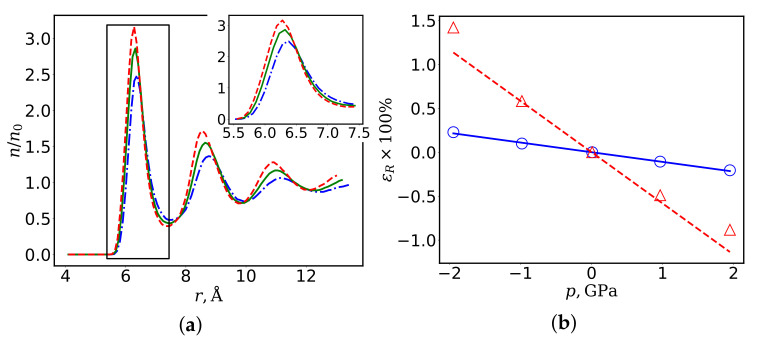
Deformation of an aluminum melt with a spherical inhomogeneity (C_60_) under uniform compression: (**a**) radial aluminum density distribution at an applied pressure p=−2 GPa (dashed-dotted curve), p=0 GPa (solid curve), and p=2 GPa (dashed curve); and (**b**) deformation of the spherical interface (the solid line and the circles, the interface coincides with the C_60_ fullerene surface; the dashed line and the triangles, along the aluminum boundary of radius *R*_1_, $n\left(R_{1}\right)/n_{0}=0.5$). The lines show the dependences obtained by approximating the MD data indicated by symbols. The inset shows the first distribution peak.

Figure 2 shows the data characterizing the deformation of the system under the action of the applied pressure. One can see from Figure 2a that the applied pressure significantly changes the radial distribution function of the dimensionless aluminum density, *n*(*r*)/*n*_0_. Here, *n*_0_ is the mean density of a homogeneous aluminum melt at zero pressure. The first maximum of the function, which characterizes the position of the aluminum boundary *R*_1_, markedly shifts under the action of the applied pressure.

The shape of the density distribution function in the region of the first maximum characterizes the position of the interface in the Al/C_60_ system. Figure 2b shows the dependences of the deformation of a spherical inhomogeneity on pressure, $\varepsilon \left(R\right)=\delta R/R_{0}$, where *R*_0_ is the radius of the inhomogeneity at zero pressure, and δR is the change in the radius under the action of the applied pressure *p*. In the first case, the fullerene radius *R* (solid line) was taken as the interface radius, and, in the second case, the radius of the aluminum boundary *R*_1_, at which the function *n*(*R*_1_) increasing from zero reaches half the average density *n*_0_. Thus, the value of *R*_1_ was calculated using *n*(*r*) by solving the equation

$$n\left(R_{1}\right)=n_{0}/2$$

in the range $0\leq R_{1}\leq r_{\max}$, where *r*_max_ defines the position of the first maximum of *n*(*r*).

One can see from Figure 2b that the deformations of the surfaces of aluminum and carbon do not coincide, with the difference between them at the boundaries of the selected pressure range being sixfold in maximum. This fact has a significant effect on the mechanical properties of an inhomogeneous medium, which in this case are determined not only by the properties of the melt and nanoparticles, but also by the relative displacement of pairs of Al–C atoms under the action of applied stresses.

The effective compressibility of the medium was calculated using the pressure and volume of the computational domain obtained in the MD simulation by the formula:

(2) $$k=-\frac{1}{V_{0}}\frac{\partial V}{\partial p}.$$

Let us assume that the dependence of the compressibility of a multicomponent system on the concentration of inhomogeneities obeys the ROM

(3) $$k=\sum_{j=1}^{N}\nu_{j}k_{j},$$

where the summation is performed over all components of the mixture; and $\nu_{j}$ and $k_{j}$ are the volume fractions and compressibility of the components, respectively. Fullerene compressibility at 1000 K was calculated in this work by the MD method and amounted to 1.41 × 10^−12^ Pa^−1^. This value is consistent with *ab initio* simulation data [17], where the compressibility was 1.15 × 10^−12^ Pa^−1^. The calculated compressibility of the aluminum melt, 24.6 × 10^−12^ Pa^−1^, corresponds to a value of 29.0 × 10^−12^ Pa^−1^ that was *ab initio* MD calculated in [39] for the aluminum melt.

**Figure 3:**
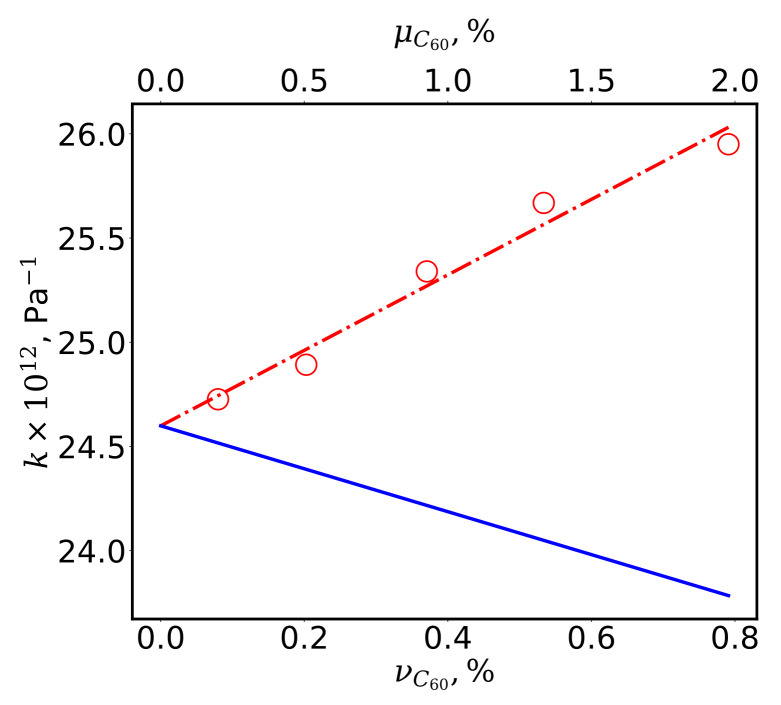
Dependence of compressibility on the concentration of fullerenes in the aluminum melt (the solid line, calculation by the ROM; the symbols, the MD data; and the dash-dotted line, the least-squares approximation of the calculated data).

Expression (3), as well as the compressibility of the components, allows us to expect a monotonic decrease in the effective compressibility of the system with increasing volume concentration of the fullerene. However, the results of the MD simulation (see Figure 3), as well as experimental data [7], indicate the opposite.

The solid line in Figure 3 shows the dependence of the compressibility on the volume $\nu_{C_{60}}$ and mass $\mu_{C_{60}}$ concentrations of fullerenes, calculated according to the ROM, and the symbols represent the results of calculations using Formula (2), where the volume and pressure were calculated by the MD method. The dashed-dotted line is the least-squares approximation of the calculated data.

## 3. Discussion

The curves of the dependence of the effective compressibility on the concentration of fullerenes, calculated by the MD method and by the ROM, demonstrate not only a quantitative but also a qualitative discrepancy. Application of the ROM does not allow one to expect an increase in the compressibility of the system with incorporated spherical particles, which are more rigid than the matrix material. Estimation of the effective inhomogeneity compressibility using the ROM (3) and linear approximation of the MD simulation results yields a value of 26.1 × 10^−12^ Pa^−1^, which is approximately 18.5 times greater than the calculated compressibility of the fullerene C_60_.

The reason for this discrepancy is the small size of the nanoparticles, which makes it impossible to neglect the interface thickness. One can see from Figure 2b that the deformations of the carbon and aluminum surfaces are incompatible, which limits the applicability of the theory of elasticity of inhomogeneous media.

For a more detailed qualitative explanation of the obtained results, we consider a simplified analytical model of a medium with a spherical inclusion. The free energy of the interface between phases 1 and 2 of a medium can be calculated by the thermodynamic integration method:

(4) $$F=\frac{1}{2}\int_{0}^{1}d\lambda \int u\left(r_{1},r_{2},\lambda \right)n_{2}\left(r_{1},r_{2},\lambda \right)dr_{1}dr_{2},$$

where *n*_2_ is a two-particle distribution function, and the value *λ* = 0 corresponds to a system whose components 1 and 2 do not interact with each other. The interaction potential between the atoms of components 1 and 2 located at the points **r**_1_ and **r**_2_, *u*(**r**_1_, **r**_2_, *λ*), provides a continuous transition from a state without interaction to a state with a real potential of interatomic interaction, *u*_0_(**r**_1_, **r**_2_):

(5) $$u\left(r_{1},r_{2},\lambda \right)=\lambda u_{0}\left(r_{1},r_{2}\right).$$

The function *n*_2_ is unknown, but it can be calculated using various numerical methods, among which the molecular dynamics simulation is considered one of the most reliable. However, in the absence of simplified analytical models, the analysis of the results of such numerical experiments is difficult.

An expression for the free energy of the interface can be derived using a simplified model widely used for the analytical description of capillarity [37,40]. We assume that carbon atoms are uniformly distributed over the nanoparticle surface. In this case, for the interatomic potential of form (1), the interaction of an aluminum atom with a nanoparticle is determined by the expression [31]

(6) $$u_{0}\left(r_{1},r_{2}\right)\approx u_{0}\left(r\right)=8\pi \epsilon \sigma^{2}n_{S_{0}}\frac{R}{r}\left[\frac{1}{10}\left(\zeta_{10}^{-}-\zeta_{10}^{+}\right)-\frac{1}{4}\left(\zeta_{4}^{-}-\zeta_{4}^{+}\right)\right],$$

where *ϵ* and *σ* are the parameters of potential (1), $n_{S_{0}}$ is the average surface density of atoms of the nanoparticle (for fullerene $n_{S_{0}}\approx 0.36$ Å^−2^), *R* is the nanoparticle radius, *r* is the distance from the center of the nanoparticle mass to the aluminum atom, and $\zeta_{m}^{\pm }={\left[\sigma /\left(r\pm R\right)\right]}^{m}$.

Note that, in the original work [31], expression (6) was obtained for a hollow particle (fullerene), but, in some cases, it can be also used for a spherical inclusion. For a spherical nanoparticle, $n_{S_{0}}$ is calculated taking into account surface atoms, while a small contribution from the interaction of internal atoms with the surrounding substance (aluminum) can be taken into account approximately in parameterizing potential (1), or completely excluded from consideration. We believe that this approximation is possible for nanoparticles with $R_{0}∼1$ nm due to a rapid decrease in potential (6) with distance *r*, and also taking into account the fact that most of the atoms of small nanoparticles are located on the surface. We can assume that this approximation is applicable for nanoparticles of size $R_{0}\gg 1$ nm with the appropriate parametrization of potential (1), in which the contribution of internal atoms to the interaction energy can be taken into account implicitly when calculating the parameters *ϵ* and *σ*; however, the limits of applicability of the model require additional studies.

Further simplification of the problem is associated with the use of the approximate aluminum density distribution function, which we equate to zero at $r<R_{1}$ and to the average density $n_{0}$ otherwise. Thus, in expression (4), the two-particle distribution function is approximated by the Heaviside step function:

(7) $$n_{2}\left(r,\lambda \right)\approx n_{0}\Theta \left(r-R_{1}\right)=\left\{\begin{array}{c} 0,r<R_{1},\\ n_{0},r\geq R_{1}, \end{array}\right.$$

where $n_{0}\approx 0.0536$ Å^−3^ is the average density of the aluminum melt, calculated in this work at 1000 K.

Assuming a temperature-independent uniform distribution of atoms, we simultaneously neglect the temperature dependence of the free energy. Therefore, these assumptions are very rough, which does not allow us to expect a high accuracy of the analytical model. The usefulness of analytical expressions lies in the possibility of a simple qualitative explanation of the results of numerical simulation, as well as in the possibility of determining the characteristic parameters of the problem for practical assessments.

The use of Expressions (6) and (7) makes it possible to perform integration of Equation (4) analytically:

(8) $$\begin{array}{c} F=w_{0}\left[\xi_{8}^{1-}-\xi_{8}^{1+}-\xi_{8}^{2-}+\xi_{8}^{2+}+\frac{8R}{9\sigma }\left(\xi_{9}^{1-}+\xi_{9}^{1+}-\xi_{9}^{2-}-\xi_{9}^{2+}\right)\right]-\\ 10w_{0}\left[\xi_{2}^{1-}-\xi_{2}^{1+}-\xi_{2}^{2-}+\xi_{2}^{2+}+\frac{2R}{3\sigma }\left(\xi_{3}^{1-}+\xi_{3}^{1+}-\xi_{3}^{2-}-\xi_{3}^{2+}\right)\right], \end{array}$$

where use is made of the notation $\xi_{m}^{i\pm }={\left[\sigma /\left(R_{i}-R\right)\right]}^{m}$; the index i=1,2 correspond to the inner and outer boundaries of the matrix; and $w_{0}$ is the characteristic energy determined by the expression

(9) $$w_{0}=\frac{2}{5}\pi^{2}\epsilon n_{0}n_{S_{0}}R\sigma^{4}.$$

The integration boundaries $R_{1}$ and $R_{2}$ correspond to the inner and outer boundaries of aluminum, which occupies the volume of a hollow sphere (for an infinite volume, $R_{2}\to \infty$). Neglecting small values of $\xi_{m}^{2\pm }$ and $\xi_{m}^{1+}$, we can obtain the final expression:

(10) $$\begin{array}{c} F_{0}=w_{0}\left(\xi_{8}+\frac{8R}{9\sigma }\xi_{9}-10\xi_{2}-\frac{20R}{3\sigma }\xi_{3}\right),\\ \xi_{m}={\left[\sigma /\left(R_{1}-R\right)\right]}^{m}. \end{array}$$

At $R_{2}-R=4\sigma$, Formulaes (8) and (10) give a difference in the calculation results within an error of 10%. This corresponds to a volume fraction of fullerenes, $\nu_{C_{60}}=1.7$%, and a weight fraction of fullerenes, $\mu_{C_{60}}=3.8$%. At these concentrations, there are usually problems with a uniform distribution of C_60_ in aluminum, and therefore $\mu_{C_{60}}$ is usually less than or equal to 2% in practice. We will assume that the compressibility of the system can be described using a mechanical model, in which, however, the inhomogeneity is characterized by certain effective values of the parameters (radius and compressibility). To ensure that the deformation compatibility conditions are met, the effective radius of the inhomogeneity is set to coincide with the cavity radius $R_{1}$. The pressure distribution for $r<R_{1}$ will be considered homogeneous. To calculate the pressure, we use the well-known thermodynamic relation [41]:

(11) $$p=-{\left(\frac{\partial F}{\partial V}\right)}_{T}.$$

Assuming that the nanoparticle volume is limited by a sphere of radius *R*_1_, we obtain

(12) $$p\approx \frac{w_{0}}{\pi R_{1}^{2}\sigma }\left[\xi_{9}+\frac{R}{\sigma }\xi_{10}-\frac{5}{2}\left(\xi_{3}+\frac{R}{\sigma }\xi_{4}\right)\right],$$

where *R* is the nanoparticle radius, which in the case of small deformations linearly depends on the pressure as

(13) $$R=R_{0}\left(1-\frac{p}{3B_{p}}\right),$$

and $B_{p}\approx 709$ GPa is the bulk modulus of a nanoparticle, calculated in this paper for the fullerene C_60_.

At zero pressure, Equation (12) yields

(14) $$R_{1,0}=R_{0}+{\left(2/5\right)}^{1/6}\sigma ,$$

which corresponds to an effective inhomogeneity radius at zero pressure.

In the case of small deformations, Expression (12) can be written in the form of a Taylor series:

(15) $$p\left(R_{1}\right)\approx -3B_{i}\frac{\delta R_{1}}{R_{1}}+O\left[{\left(\frac{\delta R_{1}}{R_{1}}\right)}^{2}\right].$$

Neglecting the terms above the first order of smallness and performing simple but rather cumbersome calculations, we obtain an approximate value of the “bulk modulus” of the interface, *B_i_*:

(16) $$B_{i}\approx 27\epsilon n_{0}n_{S_{0}}R_{0}\sigma .$$

The compressibility of an inhomogeneous medium can now be calculated using the ROM (3), where the interface volume is calculated as

(17) $$V_{i}=\frac{4}{3}\pi \left(R_{1,0}^{3}-R_{0}^{3}\right).$$

Thus, the effective value of the compressibility of a nano-inhomogeneity can be calculated by the formula:

(18) $$k_{eff}=k_{p}{\left(R_{0}/R_{1,0}\right)}^{3}+k_{i}\left[1-{\left(R_{0}/R_{1,0}\right)}^{3}\right].$$

It follows from (3) that, when a spherical nanoparticle is added to a liquid, the compressibility can be reduced only if $k_{eff}<k_{m}$, which must be taken into account when creating composite materials—for the Al/C_60_ system *k_eff_* = 31.5 × 10^−12^ Pa^−1^, and for aluminum melt *k_m_* = 24.0 × 10^−12^ Pa^−1^, which explains a monotonic increase in *k*(*ν*C_60_) obtained in the MD simulation. Hereinafter, the analytical estimates rely on such MD-calculated parameters as fullerene and aluminum-melt compressibilities.

At zero pressure acting on the liquid from the outer boundary, the pressure is $p(R_{1})\neq 0$, and hence $R_{1}\neq R_{1,0}$. To calculate $p(R_{1})$, it is necessary to estimate the value of the specific interfacial free energy.

In the expression for free energy [see Equation (4)], as the state with zero free energy, we chose the state of the melt with a cavity in it, where a spherical particle was then incorporated. The work spent on creating a spherical cavity of radius *R*_1_ can be estimated using Expression (10), where the free energy *F_c_* is calculated using the parameters $\epsilon_{M}$ and $\sigma_{M}$ of the interaction potential between matrix atoms. For aluminum, $\epsilon_{M}=0.1743$ eV and $\sigma_{M}=2.925$ Å [42]. The radius of a spherical particle “cut out” from the matrix is $R_{M}=R_{1}-{\left(2/5\right)}^{1/6}\sigma_{M}$. The free energy of the interface is expressed as

(19) $$F=F_{0}-F_{c}=F_{0}+4\pi R_{1}^{2}\gamma_{0}\left(1-\frac{\delta }{R_{1}}\right)+C,$$

where the formulae for calculating the surface tension coefficient of the matrix, *γ*_0_, and the Tolman length *δ* can be obtained directly from (10):

(20) $$\begin{array}{c} \gamma_{0}\approx 2.2\epsilon_{M}n_{0}^{5/3}\sigma_{M}^{3},\\ \delta \approx 0.27\sigma_{M}. \end{array}$$

The constant *C*, which is independent of the radius *R*_1_, will be taken equal to zero in calculations, since the expressions for physical quantities in question include only derivatives of the free energy, which can be determined up to an arbitrary constant. Given that $\delta /R_{1}∼0.1\ll 1$, we will approximately assume that the surface tension coefficient $\gamma =\gamma_{0}$ is independent of the radius *R*_1_. Assuming the pressure on the melt from the side of the boundaries to be equal to *p*, the radius *R*_1_ will be calculated by taking into account the Laplace pressure $p_{L}=2\gamma_{0}/R_{1,0}$:

(21) $$\begin{array}{c} R_{1}\left(p+p_{L}\right)=R_{1,0}\left[1-\frac{k_{eff}\left(p+p_{L}\right)}{3}\right]. \end{array}$$

To test the model, the results of analytical estimates of some characteristics of the interface were compared with the data obtained by the MD simulation. Analytical estimates were performed using the presented formulas with the above parameter values. The results of the comparison are presented in Table 1. The cavity radius *R*_1_ and the particle radius *R* were calculated at zero external pressure. In analytical estimates, the fullerene was assumed to be deformed under the action of pressure *p_L_*, and the radius *R* was calculated by Formula (13). In MD simulation, the value of the free energy *F*_0_ was replaced by the value of the potential energy of interaction between carbon and aluminum atoms. A more accurate calculation of the free energy of the interface was not performed in this work. The value of *γ*_0_ calculated by Formula (20) was compared with the data of [42], where the Lennard–Jones potential with parameters *ϵ_M_* and *σ_M_* was used to calculate the specific energies of Al (111) and (100) surfaces by the MD method.

One can see from Table 1 that, despite rather rough approximations, the analytical model makes it possible to estimate the characteristics of the interface within acceptable accuracy. The dependence of the compressibility of the medium on the concentration of fullerenes is presented in Table 2.

Compared with the MD simulation data, analytical estimates underestimate the compressibility of the medium, which is apparently due to the use of approximation (7) for the two-particle distribution function of carbon and aluminum atoms. Analysis of the data shows that the value of *k_eff_* is underestimated by about two times: the analytical estimate yields 31.5 × 10^−12^ Pa^−1^, and the approximation of the results of the MD simulation is 65 × 10^−12^ Pa^−1^. At the same time, the obtained values of *k_eff_* are more than 10 times higher than the compressibility value of the fullerene C_60_, which is *k_p_* = 1.41 × 10^−12^ Pa^−1^. The analytical model predicts an increase in compressibility with an increase in the concentration of fullerenes, and, therefore, adequately describes the nature of the dependence of the effective compressibility of the medium on the concentration of inhomogeneities.

## 4. Conclusions

We used the MD simulation to study the compressibility of a substance with nano-inhomogeneities using the example of an aluminum melt and the fullerene C_60_. The simulation indicates a decrease in the compressibility of aluminum with an increasing concentration of C_60_. This result agrees with experiment [7] but contradicts estimates using the ROM.

To explain the obtained result, the deformation of the interface during system compression was analyzed. The position of the aluminum boundary was determined from the function of the radial density distribution plotted relative to the center of mass of C_60_. A spherical surface was taken as the fullerene boundary, whose radius corresponded to the average distance from carbon atoms to the center of mass of the molecule. The analysis showed that the displacements of these boundaries under the action of an external pressure do not coincide, and the distance between them is comparable in magnitude with the fullerene radius. This fact imposes restrictions on the use of a mechanical model, which assumes the presence of a thin interfacial surface, on which the conditions of compatibility of strains and stresses can be used. In addition, the nature of the displacement of the boundaries of aluminum and fullerene reveals a possible reason for an increase in the effective compressibility of the system observed in the experiment and numerical simulation with an increase in the concentration of fullerenes.

An analytical model is proposed that takes into account the interactions between atoms of different phases at the interface and correctly predicts the nature of the change in the bulk modulus of the system with increasing nanoparticle concentration. The model relies on the formulations of the mechanics of inhomogeneous media, in which the values of the inhomogeneity parameters are replaced by effective ones that take into account the interaction of atoms of different phases. The use of analytical expressions for estimating the effective characteristics of an inhomogeneity in mechanical simulation yields an acceptable agreement with the results of MD simulations. The absence of the need for complex atomistic calculations and the simplicity of the obtained analytical expressions make the model convenient for practical estimates of the compressibility of an inhomogeneous medium.

## Figures and Tables

**Table 1:** Comparison of the results of analytical estimates of some characteristics of the Al/C_60_ interface with the data of MD simulation.

	*F*_0_, eV	*R*_1_, Å	*R*, Å	*γ*_0_, J/m^2^
MD	−9.176	5.88	3.6462	1.185 ^a^, 1.234 ^b^
Analytical estimate	−8.670	5.68	3.6519	1.42

^a,b^ are the data of [42] for Al (111) and (100) crystalline surfaces, respectively, obtained in the framework of the model of interaction between aluminum atoms, which was used in the derivation of Formula (20).

**Table 2:** Influence of the fullerene concentration on the compressibility of the aluminum melt. Comparison of analytical estimates, *k_A_*, and calculations by the MD method, *k_MD_*.

$\mu_{C_{60}}$, %	$\nu_{C_{60}}$, %	$k_{\mathrm{MD}}\times 10^{12}$, Pa^−1^	$k_{A}\times 10^{12}$, Pa^−1^
0.20	0.08	24.73	24.60
0.51	0.20	24.89	24.66
0.93	0.37	25.34	24.71
1.34	0.53	25.67	24.76
2.00	0.79	25.95	24.84

## Data Availability

The data presented in this study are available in this article.

## Abbreviations

The following abbreviations are used in this manuscript:

MD	Molecular dynamics
ROM	Rule of mixture
